# Patients' Impression of Health Care Providers' Attire in the Emergency Department

**DOI:** 10.7759/cureus.32844

**Published:** 2022-12-22

**Authors:** Imad M Khojah, Maha K Alghamdi, Bassam M Alahmari, Maria A Alzahrani, Hassan G Alshehri, Jamal S Farahat, Ghada A Merdad, Ahmed Aalam

**Affiliations:** 1 Emergency Medicine, King Abdulaziz University, Jeddah, SAU; 2 Emergency Medicine, King Abdulaziz University Hospital, Jeddah, SAU; 3 Medicine and Surgery, King Abdulaziz University, Jeddah, SAU; 4 Emergency Medicine, Prince Mohammed Bin Abdulaziz Hospital, Al Madinah, SAU; 5 Family Medicine, Johns Hopkins Aramco Healthcare, Dhahran, SAU; 6 Internal Medicine, King Abdulaziz Specialist Hospital, Altaif, SAU

**Keywords:** patients' trust in professionalism, emergency department, the attitude of a health care worker, health care providers' attire, patients' impression

## Abstract

Background

The level of patient satisfaction and, ultimately, the assessment of the quality of care are greatly influenced by physicians' capacity to leave a positive impression on patients during provider-patient interactions. The way doctors dress affects how people view their care. There have been few studies on the impact of doctors' attire on patient confidence and trust. The objective of this study is to assess patients' preferences concerning specific cultural attire and its influence on patients' trust, compliance, and perceptions of the quality of care in the emergency department.

Methods

A cross-sectional study was performed using the survey methodology for patients in emergency departments. Participants completed a written survey after reviewing doctors' portraits in different dress styles. Respondents were asked questions about the importance of the health service providers' attire in the emergency department on the patient's perception. The Statistical Package for Social Sciences version 21 (SPSS; IBM Inc., Armonk, New York) was used to perform the analysis after the data were entered into Microsoft Excel 2016 (Microsoft, Redmond, Washington). The categorical analysis was performed using the Chi-squared test to explore for relationships between the results and various variables.

Result

A self-administered questionnaire was completed by 395 patients; two responses were excluded for lack of completeness of the answers in it: 33.8%) were males (66.2%) were females (56.7%) were married (73.8%) completed university education (44.8%) were employed and (74.5%) with excellent health conditions. The questionnaire was devoted to the local setting, with pictures of the health care provider (male and female), in different types of doctor's attire included. Respondents overwhelmingly prefer male emergency physicians to dress in medical scrub (50%, p=.0001) and prefer female emergency physicians to dress in a medical scrub with a white coat (68.7%, p=.0001).

Conclusion

First impressions based on a physician's appearance serve as the foundation for assumptions about trust, confidence, and competency, particularly in circumstances when patients or family members do not already have a relationship with the provider.

## Introduction

A good doctor-patient relationship needs to have a solid basis to establish trust and enable the provision of excellent treatment. Gaining the trust of patients is challenging in order to achieve the most significant treatment outcomes [1]. Therefore, doctors are very fascinated by the most effective strategies for gaining their patients' trust. A doctor who is well-dressed may give the idea that patient contact is extremely crucial and requires preparation, whereas a doctor who is disheveled may come across as incompetent and unskilled. In fact, Hippocrates is credited with recognizing the significance of a doctor's attire on the patient-physician bond when he stated that a physician "must be clean in person, well dressed, and anointed with sweet-smelling unguents..." [2].

Previous research has studied the initial impression of the patient toward the healthcare provider's attire and how it plays a vital role in developing doctor-patient relationships. Patients will create their first impression of their doctors during the consultation, and they will be more willing to obey when they see them to be professional, empathetic, and competent, which enables them to disclose more sensitive personal information [3]. Respondents recommended traditional items for doctors, including formal wear, a white coat, and a name tag [4, 5].

In contrast, most of the patients in other studies that examined the effect of attire during patient interactions claimed that the physician's attire had no influence on their level of satisfaction and was generally an insignificant determinant [6, 7].

Nevertheless, patients' first impressions rely heavily on the healthcare provider's appearance; furthermore, multiple studies have suggested that physician attire has an important early factor of the trust, rapport, and satisfaction of the patient [1, 8, 9]. To the authors' knowledge, none of the previous studies were conducted in ED settings on a Saudi cohort. Therefore, this investigation was conducted to demonstrate the patients' impression of the healthcare providers' attire in the emergency departments.

## Materials and methods

Study design, setting, and participants

A survey-based cross-sectional study using a convenience sample methodology was conducted between February 2020 and May 2020 and targeted all populations in the Mecca region in Saudi Arabia. The Mecca region is the most populous province in Saudi Arabia, located in western Saudi Arabia, and has an extended coastline. It has an area of 153,128 km^2^ and a population of 8,557,766 as of 2017 [10]. The inclusion criteria were any adults between the ages of 18 and 65. The exclusion criteria were for participants who are under the age of 18 and over 65, and participants from outside the local community of Mecca region. Participants were accredited from within the geographical area and within the target age.

Data collection instruments

**Figure 1:**
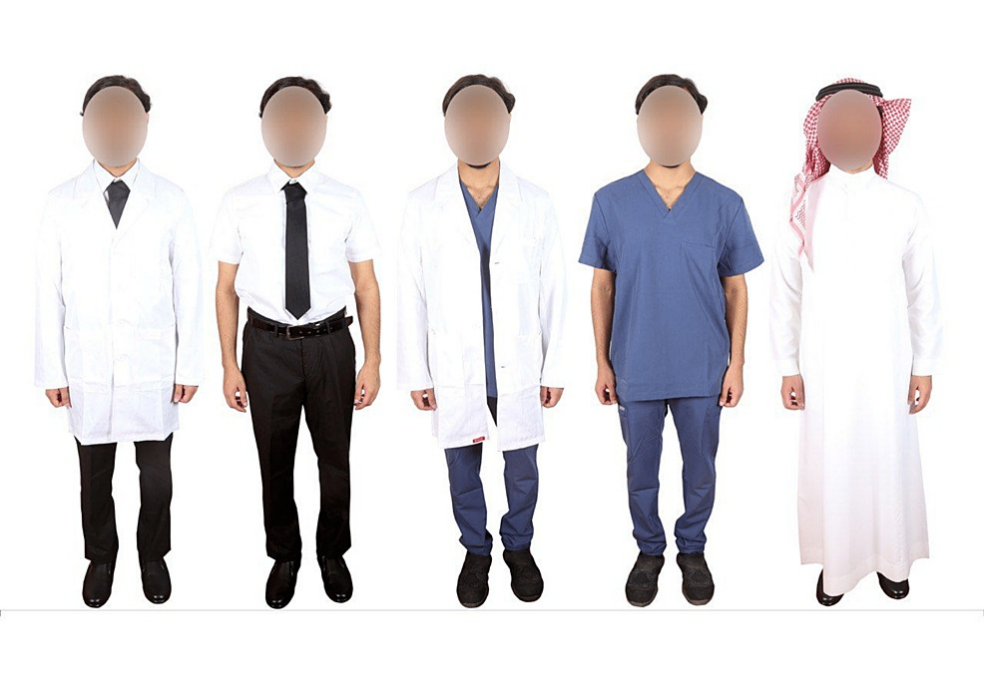
Identical male doctor wearing five different dress styles

**Figure 2:**
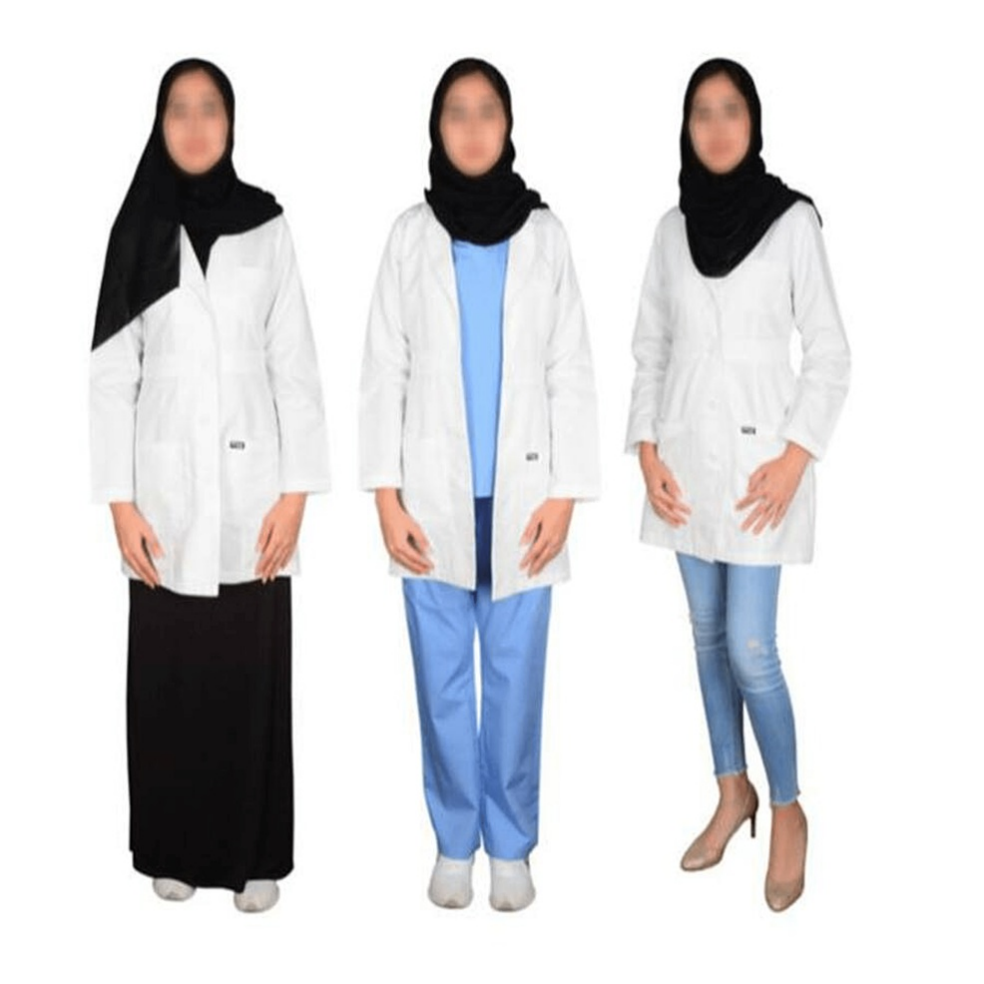
Identical female doctor wearing three different dress styles

After viewing portraits of doctors dressed in five different styles, including the traditional Saudi uniform, medical scrub, medical scrub with a lab coat, casual (tie and tuxedo pants, and tie with a lab coat for men), and three different styles for women, including jeans pants with high heels, medical scrub with a lab coat, and skirt with a lab coat, participants filled out an online survey that was distributed through social media platforms after obtaining prior approval at the beginning of the survey (Figures 1, 2). 

The researchers formulated a valid and reliable questionnaire based on previous and similar studies [11-13]. The questionnaire has a 92% reliability rate, which is highly acceptable from a statistical point of view in terms of making a high statistical study instrument usable. The value of transactions can be used consistently and acceptably for the objectives of the current study, as the reliability statistics of Cronbach's Alpha=0.92, with the number of items being 13. This outcome was found to be sufficient for using this questionnaire to gather and analyze data. The questionnaire consists of 13 questions: demographic data, the extent of physician attire preference, and the comfortability of sharing sensitive topics based on the physician's appearance.

Data entry and analysis

The data were entered into Microsoft Excel 2016 (Microsoft, Redmond, Washington) and analyzed using the Statistical Package for Social Sciences version 21 (SPSS; IBM Inc., Armonk, New York). Categorical variables are presented as frequencies and percentages. The Chi-squared test was used to analyze the categorical data to observe the association between outcomes and different variables; statistical significance was set at p-value<0.05.

## Results

Three hundred ninety-five participants have filled out and returned the questionnaire. Two responses were excluded for the lack of completeness of the answers in them. 66.2% were females, 57% were married, 73.8% had a university degree, 46.8% were employees, and 74.6% were in good health. The characteristics of the study participants are listed in Table 1.

**Table 1:** Characteristics of study participants (n=393)

Characteristic		Value (%)
Gender	Female	66.2
	Male	33.8
Marital status	Married	57
	Non-married	43
Education	Finished university education	73.8
	Did not finish university education	26.2
Employment status	Employed	46.8
	Non-employed	53.2
Health status	Having good health	74.6
	Not having good health	25.4

**Figure 3:**
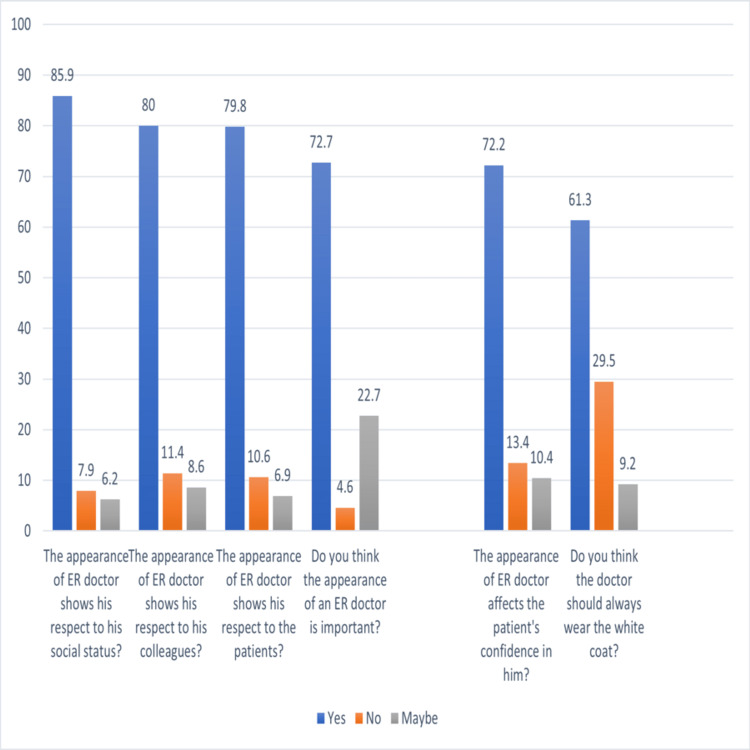
Results of physician attire questionnaire

The extent of the preference regarding the physician's attire is seen in Figure 3.

The results show that (85.9%) of the respondents think that the appearance of an emergency physician shows his respect for his social status, (80%) of the respondents think that the appearance of an emergency physician shows his respect for his colleagues, (79.8%) of the respondents think that the appearance of an emergency physician shows his respect for the patients, and (72.7%) of the respondents also answered that they think the appearance of an emergency physician is important (p-value<0001).

According to Chi-squared tests in Table 2 and Table 3, the preference patterns for male and female doctors were all highly significant difference from an equal choice among the various dress styles (p-value<0.0001). The majority of respondents (50%) prefer medical scrub for male doctors, followed by medical scrub with a white coat (25%), casual (20.3%), neutral (4.6%), and traditional dress (0.1%) on average across all preference questions. For female emergency doctors, the most common attire is a medical scrub with a white coat (68.7%), followed by a long skirt with a white coat (19.3%), jeans (6.1%), and neutral (5.9%). Respondents' trust and confidence were significantly associated with a preference for professional attire (p-value<.0001).

**Table 2:** Preferred attire for the male physicians

	Attire of preferred for male emergency physician					p-value
	Scrubs with a white coat (%)	Saudi traditional dress (%)	Casual (%)	Scrubs (%)	Does not matter (%)	
Which one would you prefer an emergency physician wear?	25	0.1	20.3	50	4.6	< .0001

**Table 3:** Preferred attire for the female physicians

	Attire of preferred for female emergency physician				p-value
	Scrubs with a white coat (%)	Long skirt with a white coat (%)	Jeans (%)	Does not matter (%)	
Which one would you prefer an emergency physician wear?	68.7	21.9	6.1	5.9	< .0001

## Discussion

In this study, respondents greatly preferred wearing professional clothing, with white coats for female doctors and scrubs for male doctors. The results of our study are similar to those of many other studies conducted around the world in a variety of settings [3, 11, 14], except that none of the studies were conducted in the setting of an emergency department. This study indicated that professional attire was associated with the appearance of an emergency physician showing his or her respect for the social status; 80% of the respondents think that the appearance of an emergency physician shows his or her respect for the staff, and 79% of the respondents think that the appearance of an emergency physician shows his or her respect for the patients. This is consistent with research by McNaughton-Filion et al., who found that a patient's level of trust in a doctor is significantly influenced by the doctor's choice of dress [15]. 

In this study, 393 participants were surveyed about emergency doctors' attire, which revealed that respondents preferred scrubs for male emergency physicians and scrubs with a white coat for female emergency physicians. The respondents' variety of cultural backgrounds can be one of the possible explanations for this finding, in addition to the impression the patients receive from the physician's appearance and clothing choices.

Al-Ghobain (2012) [11] addressed the issue of Saudi local attire in his research of outpatient clinics, which revealed that most Saudi patients prefer a Saudi doctor to dress formally and that only 9.7% of patients prefer Saudi national attire. Both results are similar, whereas, in a family medicine practice, the Saudi national attire was more noticeable (26.3%). This may help to explain how a patient's preference for certain attire changes depending on the specialty and clinical setting. 

In a different study carried out in outpatient surgical clinics, respondents' perceptions of the care they were given were not significantly influenced by the surgeons' choice of dress, and the respondents had no preferences for white coats or more standard surgical attire [14]. This could explain the surgeons' primary role towards their patients in the operating room, where they are sedated and unaware of the scrubbed surgeon; additionally, surgeries should be performed in an aseptic environment, and white lab coats play no role in that. Additionally, many other research, including Toquero's (2011) study, stated that patients may still not prefer their doctor to dress in a particular way [16]. An earlier study conducted by Kurihara et al. in 2014 discovered that a patient's capacity to trust a doctor was significantly influenced by the way a doctor dressed [17].

The current study data suggests that doctors, who are dressed in professional clothes, are associated with a positive impression about the level of positive patient behaviors and professional doctors. In this study, there were some interesting results regarding the appearance of doctors. The participants gave much more importance to the appearance of the doctor in uniform because it gives the impression of his/her respect for social relations, and it seems that patients have more confidence in doctors who wear more formal clothing and maintain a professional appearance than doctors who don't. 

The limitation of our research is that the study took place only in emergency departments in the Mecca region. Therefore, the preferences of the patients might not be representative of all Saudi respondents. More research in different regions of Saudi Arabia, as well as a diverse audience beyond cultural boundaries, are required. Moreover, not all demographic features of the study participants have been addressed, including religion and economic status, which influence perceptions of medical professionals.

## Conclusions

The results of the current study confirm that patients prefer the health care providers in emergency departments to be in formal attire, and it plays an important role in influencing trust and confidence-building between the patient and the physician.

## References

[1] Chung H, Lee H, Chang DS, Kim HS, Lee H, Park HJ, Chae Y (2012). Doctor's attire influences perceived empathy in the patient-doctor relationship. Patient Educ Couns.

[2] Chang DS, Lee H, Lee H, Park HJ, Chae Y (2011). What to wear when practicing oriental medicine: patients' preferences for doctors' attire. J Altern Complement Med.

[3] Rehman SU, Nietert PJ, Cope DW, Kilpatrick AO (2005). What to wear today? Effect of doctor's attire on the trust and confidence of patients. Am J Med.

[4] Keenum AJ, Wallace LS, Stevens AR (2003). Patients' attitudes regarding physical characteristics of family practice physicians. South Med J.

[5] Cha A, Hecht BR, Nelson K, Hopkins MP (2004). Resident physician attire: does it make a difference to our patients?. Am J Obstet Gynecol.

[6] Ikusaka M, Kamegai M, Sunaga T, Narita N, Kobayashi H, Yonenami K, Watanabe M (1999). Patients' attitude toward consultations by a physician without a white coat in Japan. Intern Med.

[7] Neinstein LS, Stewart D, Gordon N (1985). Effect of physician dress style on patient-physician relationship. J Adolesc Health Care.

[8] Bianchi MT (2008). Desiderata or dogma: what the evidence reveals about physician attire. J Gen Intern Med.

[9] Brandt LJ (2003). On the value of an old dress code in the new millennium. Arch Intern Med.

[10] Mecca Province (2022). Mecca province. https://en.wikipedia.org/wiki/Mecca_Province.

[11] Al-Ghobain MO, Al-Drees TM, Alarifi MS, Al-Marzoug HM, Al-Humaid WA, Asiry AM (2012). Patients' preferences for physicians' attire in Saudi Arabia. Saudi Med J.

[12] Batais MA (2014). Patients' attitudes toward the attire of male physicians: a single-center study in Saudi Arabia. Ann Saudi Med.

[13] Hueston WJ, Carek SM (2011). Patients' preference for physician attire. Fam Med.

[14] Edwards RD, Saladyga AT, Schriver JP, Davis KG (2012). Patient attitudes to surgeons' attire in an outpatient clinic setting: substance over style. Am J Surg.

[15] McNaughton-Filion L, Chen JS, Norton PG (1991). The physician's appearance. Fam Med.

[16] Toquero L, Aboumarzouk O, Owers C, Chiang R, Thiagarajah S, Amin S (2011). Bare below the elbows - the patient's perspective. WebmedCentral.

[17] Kurihara H, Maeno T, Maeno T (2014). Importance of physicians' attire: factors influencing the impression it makes on patients, a cross-sectional study. Asia Pac Fam Med.

